# Dataset on c-Fos expression within components of corticostriatal thalamocortical circuits during the expression of a compulsive-like behavior in the female rabbit: Brain-behavior relationships

**DOI:** 10.1016/j.dib.2020.106696

**Published:** 2020-12-30

**Authors:** Hugo Cano-Ramírez, Lorena Paola Pérez-Martínez, Kurt L. Hoffman

**Affiliations:** Centro de Investigación en Reproducción Animal (CIRA), Universidad Autónoma de Tlaxcala-CINVESTAV, Mexico

**Keywords:** Compulsive behavior, c-Fos immunohistochemistry, Rabbit, Prefrontal cortex, Striatum, Midline thalamus, Exploratory Factor Analysis

## Abstract

The dataset describes regional brain c-Fos expression and a component of maternal nest building behavior (“straw carrying”) in 5 late term pregnant rabbits that had been allowed to interact with straw (a nest building material) for a discrete period (30 min), during which repetitive straw carrying behavior was initiated. Animals were sacrificed for brain c-Fos immunoreactivity 1 h after straw was placed into their cage. *Regional brain c-Fos expression:* Neuronal c-Fos expression is known to associate with a sustained increase in neuronal excitation above resting levels, primarily due to its induction in response to increased glutamatergic input and corresponding activation of the NMDA receptor. In practice, c-Fos expression is taken to be an indication of an increase in “neuronal activity”. Importantly, there is a lag of approximately 20 to 30 min between the onset of the stimulus that caused increased excitation, and the initiation of neuronal c-Fos expression, and c-Fos has a cellular half-life of approximately 1 h. Thus, the pattern of brain c-Fos expression within a brain histological section represents a composite snapshot of “superimposed” regional activations that occurred within approximately 30 min to 2 h prior to sacrifice. *Behavioral variables:* Behavioral variables included in the present dataset are those that reflect the repetitive nature of straw carrying (straw carrying cycle frequency), as well as individual subcomponents of this behavior (collecting straw, interacting with the nest site), and indicators of the “rigidity” of expression of these subcomponents across all cycle repetitions (standard deviations of time spent collecting straw, time spent interacting with nest site). Exploratory Factor Analysis (EFA) with cluster rotation was applied in an exploratory manner in order to clarify correlational relationships between regional c-Fos expression and specific behavioral variables.

## Specifications Table

**Table:** 

Subject	Behavioral Neuroscience
Specific subject area	The present data were collected as part of an exploratory study to characterize regional brain activity associated with the performance of the “straw carrying” phase of maternal nest building in the rabbit, which has been proposed as a possible animal model for defining neurobiological control mechanisms for compulsive-like behavior. The main objective of this line of research is to compare regional brain activity in the nest-building rabbit to regional brain activity associated with human obsessive-compulsive disorder, as determined from brain imaging techniques.
Type of data	Table Analysed
How data were acquired	Light Microscope (Olympus CH2), brightfield optics ImagePro version 5.1 JASP 0.11 statistical software (https://jasp-stats.org)
Data format	Raw Analyzed
Parameters for data collection	Data were collected from female rabbits, on day 28 of pregnancy. Nest material was provided for 30 min. c-Fos immunolabelled brain tissue sections were selected pseudo-randomly. Sampled areas of fixed dimensions were defined based on unambiguous and replicable tissue landmarks. C-Fos labelled cells were automatically identified and counted based on label size and color using ImagePro version 5.1 software.
Description of data collection	Data were acquired as part of an ongoing project that explores similarities in the neurobiological substrates of obsessions-compulsions and maternal nest building behavior in the rabbit. The latter is an adaptive, innate behavior that involves repetitive components expressed in a rigid manner. The present data were derived from 5 pregnant rabbits that were performing maternal nest building behavior. Data were collected as described in the methodology section, and were compiled into an Excel spreadsheet and later imported into JASP software for statistical analysis. All values were rounded to whole numbers before statistical analysis.
Data source location	Centro de Investigación en Reproducción Animal (CIRA), Universidad Autónoma de Tlaxcala-CINVESTAV, Tlaxcala, Tlaxcala Mexico
Data accessibility	Mendeley http://dx.doi.org/10.17632/kfb3fw92rh.1
Related research article	H. Cano-Ramírez, K.L. Hoffman, Activation of cortical and striatal regions during the expression of a naturalistic compulsive-like behavior in the rabbit, Behav Brain Res. 351 (2018) 168-177.https://doi:10.1016/j.bbr.2018.05.034 H. Cano-Ramírez, K.L. Hoffman, Activation of the orbitofrontal and anterior cingulate cortices during the expression of a naturalistic compulsive-like behavior in the rabbit, Behav. Brain Res. 320 (2017) 67–74. https://doi:10.1016/j.bbr.2016.11.022

## Value of the Data

• The present data are useful for revealing associations between regional brain activity (c-Fos expression) within cortico-striatal circuits and the performance of specific subcomponents of a compulsive-like behavior.
• These data may be useful to those studying cortico-striatal circuits and their role in modulating compulsive-like behavior, those studying the neurobiology of maternal behavior, and those studying neural control of innate, species specific behaviors.
• Possible functional relationships between regional brain activity and the performance of compulsive-like, maternal, and/or species specific behaviors can be derived from the present data. For example, EFA revealed a possible positive association between midline thalamus activity and frequency of repetition of this compulsive-like behavior.

## 1 Data Description

Data were acquired as part of an ongoing project that explores similarities in the neurobiological substrates of human obsessive-compulsive symptoms and maternal nest building behavior in the rabbit. Although the latter is innate and adaptive, it has some important characteristics of a compulsion: it is highly motivated, executed in a rigid almost stereotypical manner, yet is focussed on a particular goal and therefore retains some flexibility in its expression (definition of *compulsion* derived from [1]). The present line of study is motivated by the premise that a detailed understanding of neurobiological mechanisms that initiate, maintain, and terminate nest building in the rabbit should generate hypotheses on mechanisms that may be dysfunctional in human obsessive-compulsive disorders. Since hypotheses for obsessive-compulsive symptoms propose that these symptoms arise from dysfunction in brain mechanisms that either initiate or stop specific repetitive behavioral patterns [2], we are particularly interested in neurobiological mechanisms that engage this repetitive behavior as well as those that stop it [3]. The present data were derived from 5 pregnant rabbits that had initiated maternal nest building behavior 30 min before being sacrificed. C-Fos data from prefrontal cortex and striatum from these 5 rabbits were analyzed independently and in a distinct manner (treatment group comparisons) in Cano-Ramírez and Hoffman (2017 and 2018, respectively). The present dataset comprises these c-Fos prefrontal and striatal data along with unpublished c-Fos data from the midline thalamus and posterior hypothalamus of these same rabbits, in addition to unpublished behavioral data. Moreover, in the present study, these data were analyzed in a novel manner (data-driven, exploratory factor analysis). We limited the time that the rabbits were engaged in nest building to 30 min, because our objective was to visualize brain activity associated with the initiation and early engagement of this behavior, rather than activity that may be associated with its termination. Data were collected as described in the Methodology section, and were compiled into an Excel spreadsheet and later imported into a JASP spreadsheet for statistical analysis. All values were rounded to whole numbers before statistical analysis.

### 1.1 Explanation of spreadsheet

The Excel spreadsheet contains mean c-Fos cell density (a measure of relative c-Fos expression) of various regions of the frontal cortex, parietal cortex, striatum, and midline thalamus, as well as a number of behavioral measures corresponding to the animal's activity during the time in which c-Fos expression was induced. Following is an explanation of each of the spreadsheet columns.

a. Rabbit ID. Identifications for each individual were assigned at birth. New breeder females that entered the colony were assigned an identification letter, for example, “C”. Each breeder female was mated with three different males, chosen at random and according to availability at the time of mating. The offspring were subsequently identified with the mother's identification, plus a number. Therefore, rabbit ID “C1, C2, C3” would correspond respectively to individuals 1, 2, and 3 born to female rabbit “C”. Here, rabbit ID C14 refers to individual 4 born to female C1. Thus, it can be seen that the 5 individuals included in this dataset were derived from distinct maternal lineages.
b. LatEnter: Latency (in seconds) to enter nest box while carrying straw. Straw was placed into the cage at t = 0 s. Typically, the pregnant female rabbit quickly approaches the straw and begins to nibble on it. Very shortly (seconds) thereafter, she begins to make stereotyped head bobbing motions (which in nature presumably facilitate the extraction of dry grass from the soil substrate), while gathering pieces of straw in her mouth. Then, the female stops collecting and returns to the nest box and enters it while carrying the straw in her mouth. Thus, the value of the “Lat enter” variable is a function of the latency to begin collecting straw as well as the duration of straw collecting before returning to the nest box.
c. Cycles: The total number of straw carrying cycles completed across t =  0–30 min. One complete straw carrying cycle was defined as the following behavioral sequence: Collecting straw in mouth, carrying straw back to nest box and entering the nest box with straw in mouth, and exiting the nest box. While inside the nest box, the rabbit may vigorously scratch the floor of the nest box (“digging”; see variables h, i, j, k), and/or drop the straw from her mouth onto the floor of the nest box (“deposit”; see variable # l, m, n). After exiting the nest box, the rabbit may either begin collecting straw again (thus beginning a new cycle) or not resume collecting.
d. Cycles15: The number of straw carrying cycles completed across t = 0–15 min.
e. Cycles30: The number of straw carrying cycles completed across tt = 16–30 min.
f. InsideMean: The mean time (in sec) during the straw carrying cycle that was spent inside the nest box. Each cycle comprises a phase in which the rabbit is inside the nest box: from the moment she enters the nest box carrying straw, to the moment she exits the nest box. For each cycle, this duration was recorded, and the mean and standard deviation of these durations across all cycles was calculated.
g. InsideSD: The standard deviation (in sec) of the mean time spent inside the nest box. This variable is considered to be an indication of “inflexibility”, or “rigidity” of the rabbit's behavior. Low SD values would indicate that the rabbit very rigidly – with respect to allocation of time – performed each phase of the straw carrying cycle. Conversely, higher SD values would indicate greater flexibility in the manner in which the female allocated her time to each phase of the cycle.
h. LatDig: Latency (in seconds) to vigorously scratch (“dig”) onto the floor of the nest box.
i. Dig: Number of cycles in which the rabbit “dug” onto the floor of the nest box across t = 0–30 min.
j. Dig15: Number of cycles in which the rabbit “dug” onto the floor of the nest box across t = 0–15 min.
k. Dig30: Number of cycles in which the rabbit “dug” onto the floor of the nest box across t = 16–30 min.
l. Deposit: Number of cycles in which the rabbit deposited straw inside the nest box, across t = 0–30.
m. Deposit15: Number of cycles in which the rabbit deposited straw inside the nest box, across t = 0–15.
n. Deposit30: Number of cycles in which the rabbit deposited straw inside the nest box, across t = 16–30.
o. OutsideMean: The mean time (in sec) during the straw carrying cycle in which the rabbit spent outside the nest box. Although not explicitly registered as such, this variable for the most part reflects the duration of straw collecting during a given cycle.
p. OutsideSD: The standard deviation (in sec) of the mean time spent outside the nest box.
q. CycleMean: The mean duration (in sec) of the straw carrying cycles across t = 0–30 min.
r. Cycle SD: The standard deviation (in sec) of the mean cycle duration.
s. InsideMax: Maximum duration that the rabbit spent inside the nest box during a straw carrying cycle.
t. ThalPVd: Density of c-Fos-positive cells (# c-Fos positive cells / mm2) of the bilateral paraventricular thalamic nucleus (PV).
u. ThalPVv: Density of c-Fos-positive cells of the ventral paraventricular thalamic nucleus (PVv).
v. ThalRhRe: Density of c-Fos-positive cells in the ventral midline (immediately dorsal to the third ventricle), designated in the present study as the Reuniens/Rhomboid región (Re/Rh).
w. pHYPd: Density of c-Fos-positive cells in the dorsal region of the posterior hypothalamus (pHYPd).
x. pHYPv: Density of c-Fos-positive cells in the ventral region of the posterior hypothalamus (pHYPv).
y. ACC: Density of c-Fos-positive cells in the anterior cingulate cortex.
z. OFC: Density of c-Fos-positive cells in the orbitofrontal cortex.
aa. PL: Density of c-Fos-positive cells in the prelimbic cortex.
bb. IL: Density of c-Fos-positive cells in the infralimbic cortex.
cc. Premotor: Density of c-Fos-positive cells in the premotor cortex.
dd. Piriform: Density of c-Fos-positive cells in the piriform cortex.
ee. dCaud: Density of c-Fos-positive cells in the dorsal caudate.
ff. mCaud: Density of c-Fos-positive cells in the medial caudate.
gg. vCaud: Density of c-Fos-positive cells in the ventral caudate.
hh. dPut: Density of c-Fos-positive cells in the dorsal putamen.
ii. mPut: Density of c-Fos-positive cells in the medial putamen.
jj. vPut: Density of c-Fos-positive cells in the ventral putamen.
kk. Motor: Density of c-Fos-positive cells in the primary motor cortex.
ll. Somatosensory: Density of c-Fos-positive cells in the primary somatosensory cortex.

### 1.2 Exploratory Factor Analysis (EFA) with cluster rotation (JASP 0.11 software)

In order to explore relationships between regional c-Fos expression (a marker for neuronal activity; [4]) and the various behavioral components of the “straw carrying” phase of maternal nest building in the rabbit, we performed EFA with cluster rotation on this sample of 5 rabbits. According to de Winter and colleagues [5], considerations for performing EFA on small sample sizes include: 1) the number of extracted factors should not be large (in the present analysis, we extracted 2 to 4 factors); 2) the number of variables should be large (our analysis included 23 histological and behavioral variables); and the factor loadings should be strong (in our analysis, most ranged from 0.7 to 1.0). In the present study, the resulting 3-factor solution was both consistent with known brain circuits (limbic, associative, and sensorimotor corticostriatal processing areas loaded onto distinct factors), and implicated novel areas that clustered with frequency of behavioral repetition (midline thalamus and posterior hypothalamus).

For the EFA, we considered all brain region variables, along with a subset of the behavioral variables that were most relevant for our research objectives. These behavioral variables were: 1) Cycles15, representing repetitive straw carrying behavior expressed during the initial interaction with straw; 2) Cycles30, representing repetitive straw carrying behavior during the last half of the observation period; 3) OutsideMean, a measure of average time spent collecting straw; 4) InsideMean, a measure of average time spent interacting with the nest site (e.g., manipulating the nest material inside the nest box); 5) InsideMax, maximum time spent interacting with the nest site. Of the behavioral variables included in the dataset, these best represent quantitatively the repetitive nature of the behavior (cycle frequency), interaction with the provoking stimulus (straw collecting) and goal monitoring (the status of completion of the straw nest). We selected the option to display only those factor loadings greater than 0.50. We examined 2-, 3-, and 4-factor solutions (see supplementary materials for complete analysis).

**Fig. 1:**
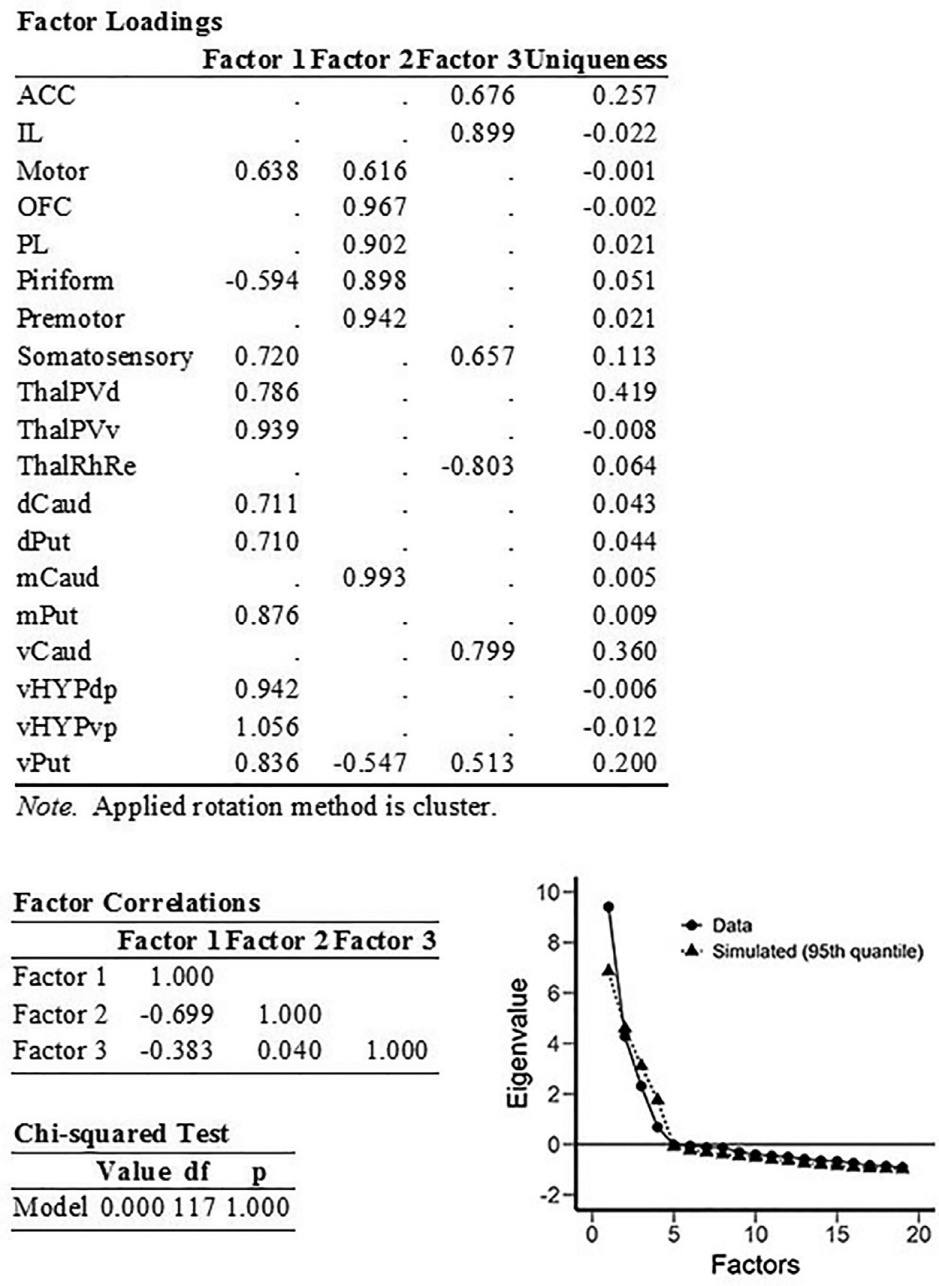
EFA of data on regional c-Fos expression, 3-factor solution with cluster rotation. Factor correlations, results of Chi-squared test, and scree plot are shown. Factor loadings onto each of the 3 factors are shown for each of the variables, along with each variable's Uniqueness (the proportion of variance that is not accounted for by the 3-factor solution). Factor 1 encompassed sensorimotor processing regions (primary somatosensory cortex, putamen, primary motor cortex and dorsal caudate-putamen); Factor 2 encompassed association processing regions (dorsolateral and orbitofrontal cortex, medial caudate); and Factor 3 encompassed limbic processing regions (ACC, IL, ventral caudate-putamen), and included a strong negative loading corresponding to the ventral midline thalamus (region of the Reuniens/Rhomboid nuclei).

**Fig. 2:**
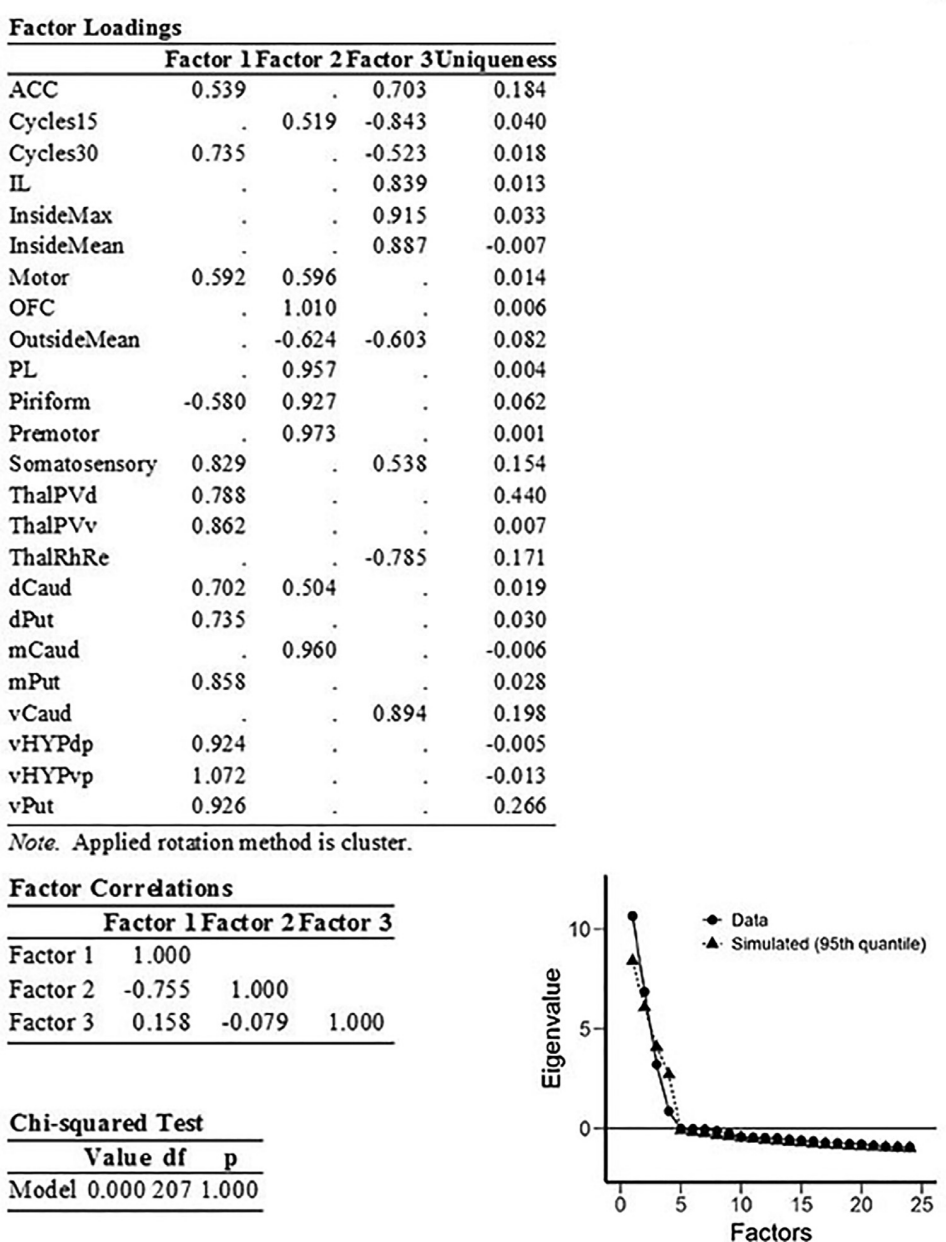
EFA applied to regional brain c-Fos expression and selected behavioral variables. Factor correlations, results of Chi-squared test, and scree plot are shown. Cycles15 loaded negatively onto Factor 3 (limbic), while Cycles30 loaded positively onto Factor 1 (sensorimotor). Midline thalamus c-Fos labelling was positively correlated with frequency of straw carrying cycles.

**Fig. 3:**
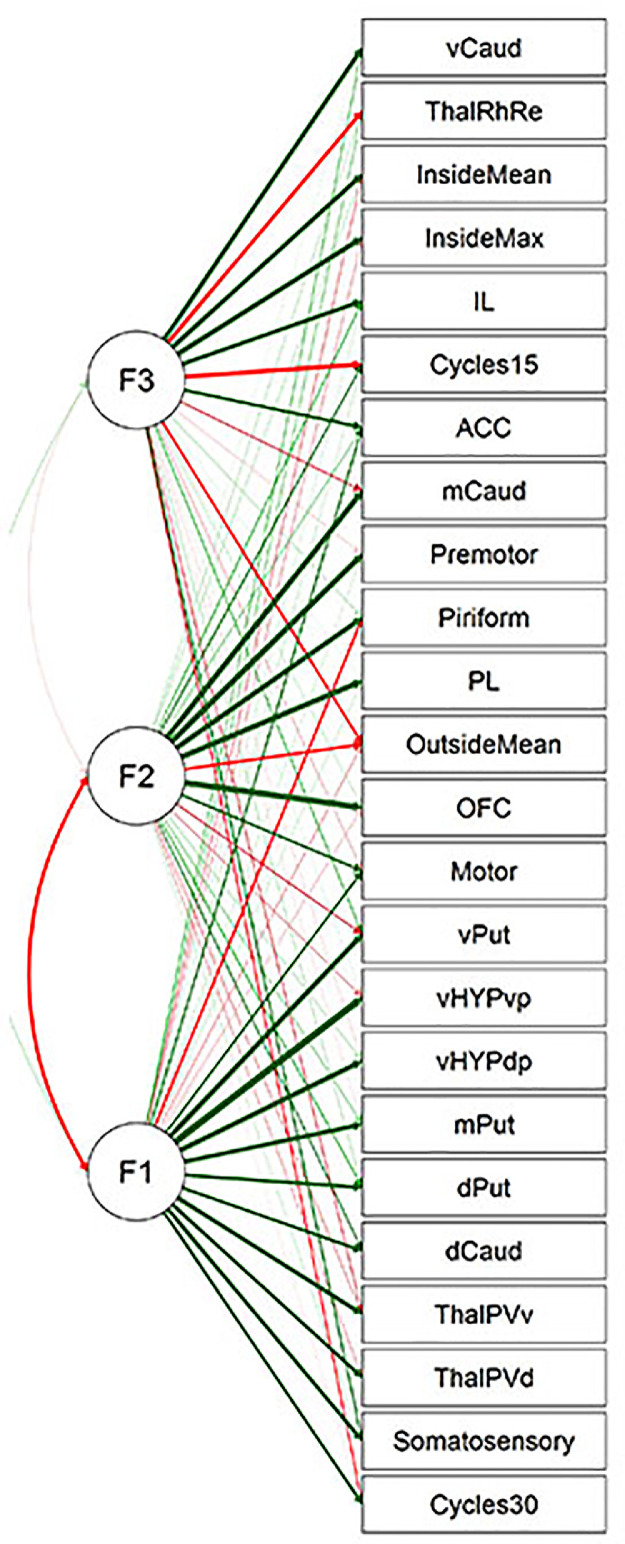
Path diagram corresponding to the 3-factor solution of EFA, cluster rotation. Green and red arrows denote positive and negative factor loadings, respectively. Thickness of arrows represents strength of loading.

**Fig. 4:**
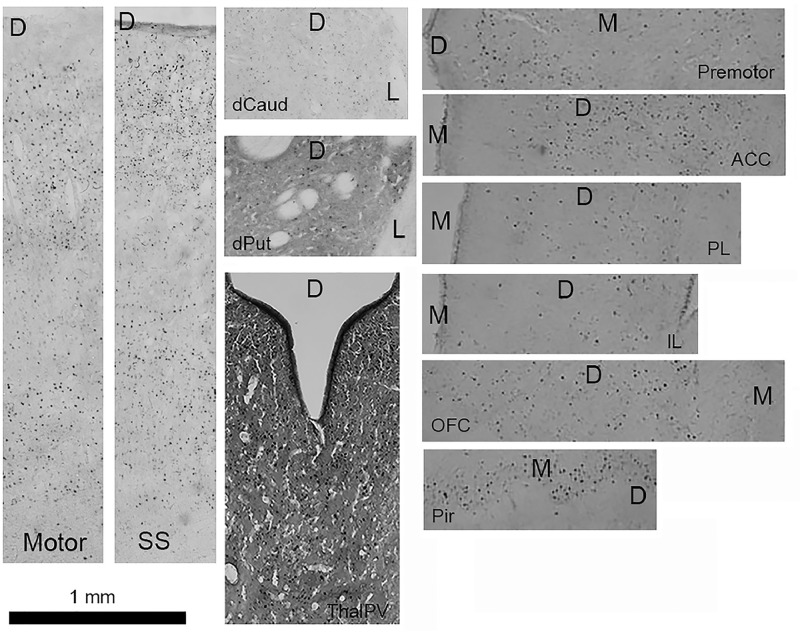
c-Fos immunolabeling of representative sections of motor and somatosensory (SS) cortices, dorsal caudate (dCaud), dorsal putamen (dPut), dorsal midline thalamus (paraventricular nucleus; ThalPV), premotor cortex, anterior cingulate cortex (ACC), prelimbic (PL), infralimbic (IL), orbitofrontal cortex (OFC), and piriform cortex (Pir). D=dorsal, M=medial, L=lateral.

First, we entered into the EFA only data on c-Fos expression (Fig. 1). In a second analysis, we entered brain region c-Fos data along with behavioral variables that we considered the most relevant for the context of modelling compulsive-like behavior (Fig. 2). Finally, we generated a path diagram to illustrate the nature and magnitude of relationships among these variables, considering a 3-factor solution (Fig. 3). Fig. 4 shows representative sections of c-Fos label from several of the representative brain regions that we analyzed.

## 2 Experimental Design, Materials and Methods

### 2.1 Animals

Data from 5 pregnant female rabbits are presented. Each female rabbit had been mated with a male, built a maternal nest with straw and hair, and had given birth at least twice (were “multiparous”) prior to entering the experiment. The multiparous female rabbits of the present dataset were mated with 2 or 3 sexually experienced males (pregnancy day 0; P0), and placed into a large cage (90 cm long × 60 cm wide × 40 cm high) containing a wooden nest box 49 cm × 28 cm × 27 cm) with an entrance hole (22 cm diameter) cut into the front. They were maintained on a 14:10 h light:dark cycle, at ambient temperature (11–26 degrees C). Food and water were given ad libitum, but straw was not placed into the cage until the initiation of the experiment (t = 0 min, day 28 of pregnancy; P28).

### 2.2 Experimental procedure

#### 2.2.1 Behavioral testing and perfusion

In the morning of day P28, straw was placed into the cage, but outside of the nest box (at t = 0 min), and the rabbit's behavior was videorecorded for the following 60 min. At t = 30 min, all straw was removed from the cage and nest box. At t = 60 min, the rabbit was anesthetized an intramuscular injection of xilazine and ketamine, and then given a intravenous overdose of pentobarbital. The rabbit was immediately perfused with approximately 1–2 L saline solution (0.9% NaCl), followed by 1–2 L paraformaldehyde solution (4%). The animal was decapitated, and the head was stored overnight at room temperature. The brain was dissected from the skull, and cut coronally into 4 blocks, which were then placed into a solution of 10% sucrose/0.1 M PO4 buffer, and kept at 4 degrees C. On the following day, the solution was replaced with 20% sucrose/0.1 M PO4 buffer. When the tissue blocks sank to the bottom of the solution (indicating that equilibrium of sucrose concentration had been reached between tissue and solution; usually after 1–2 days), the solution was changed to 30% sucrose/0.1 M PO4 buffer. When equilibrium was reached between tissue and sucrose solution, the tissue blocks were removed from the solution and frozen at -20 degrees C, and stored at that temperature until sectioning.

#### 2.2.2 Brain sectioning and immunohistochemistry

The brains were sectioned in a cryostat (40 μm; Leica CM1860) starting at the rostral tip of the frontal lobe and continuing through the thalamus. The sections were stored in a solution of cryoprotectant at -20°C. For c-Fos immunohistochemistry, sections encompassing the regions of interest were selected and washed three times in phosphate buffer (PB) 0.1 M, pH 7.4 and then for 10 min in 0.05%. After preincubation for 1 h in 1% normal donkey serum (Santa Cruz Biotechnology,Santa Cruz, CA) sections were incubated for 48 h at 4°C in the polyclonal antibody SC-52-G (Santa Cruz Biotechnology), diluted at 1:2000 with 0.1% Triton X-100 (Sigma, St. Louis, MO) in 0.1 M PB. The antibody solution was removed and sections were washed 3 times in 0.1 M PB, and then processed with the Vectastain Elite ABC kit, Vector Laboratories, Burlingame, CA (biotinylated donkey anti-goat serum IgG, 1 h at room temperature (RT); avidin and biotinylated horseradish peroxidase solution, 1 h at RT). Sections were rinsed 3 times in 0.1 PB, and then incubated in a diaminobenzidine/nickel substrate working solution for 5 min (DAB peroxidase substrate SK-4100; Vector Laboratories). Sections were mounted onto polylysine coated slides, dehydrated and then cover-slipped with a histological mounting medium.

#### 2.2.3 Sampling of brain regions for c-Fos analysis

A long-term objective of this line of research is to understand how cortico-basal ganglia-thalamocortical circuits modulate the initiation, maintenance, and termination of straw-carrying behavior. Therefore, we selected as areas of interest the medial prefrontal cortex and its striatal target (the ventral caudate), dorsal and lateral prefrontal cortex and their striatal target (medial caudate), and primary sensory and motor cortex and their striatal target (dorsal caudate and putamen). Midline thalamus and posterior hypothalamus were identified in preliminary c-Fos experiments as regions strongly associated with straw carrying behavior (unpublished observations), and are known to receive afferents from the medial prefrontal cortex. Specifically, the following brain regions were considered: anterior cingulate cortex (ACC; BA 24), prelimbic area (BA 32), infralimbic area (BA 25), premotor area (BA 8), piriform cortex, orbitofrontal cortex (OFC), somatosensory cortex, primary motor cortex, caudate, putamen, and midline thalamus. The ACC, prelimbic, infralimbic, and premotor cortices were identified according to Buchanan and colleagues [6]. The OFC was defined as the dorsal bank of the olfactory sulcus, based on neuroanatomical studies of the rabbit, as well as through extrapolation from the rat literature [7,8]. In the rabbit (unlike the rodent), the striatum is divided by the internal capsule into the caudate nucleus and putamen [9]. The primary somatosensory and motor cortices were identified based on previous descriptions of the rabbit neocortex [10,11]. The somatosensory area sampled would encompass the lip region, while the primary motor cortex region sampled (area Precentral 1, Prc1 is most likely associated with the “trunk, eye muscles, and rhinarium”). This region is medially adjacent to regions of S1 that correspond to the forepaws. The midline thalamus was chosen for analysis due to its known afferents originating from the medial frontal cortex (ACC, PL, IL) in the rabbit [6]. Midline thalamic regions were delineated based on unambiguous tissue landmarks (i.e., the lateral and third ventricles, the midline); no attempt was made to delineate histological boundaries of specific midline nuclei (e.g., the Rhomboid or Reuniens), as these are relatively ambiguous.

*Photomicrographs of prefrontal cortex.* Two independent coronal sections were selected pseudo-randomly to be photographed. These sections corresponded to the following coronal orientation planes of the Brain Atlas of the Domestic Rabbit (Oryctolagus cuniculus) (University of Wisconsin-Madison Comparative Mammalian Brain Collection website; http://neurosciencelibrary.org/Specimens/lagomorpha/domesticrabbit/sections/thumbnail.html): #280 (representing the OFC) and #320 (representing the ACC, prelimbic area, infralimbic area, premotor area, and piriform cortex).

*Photomicrographs of the striatum and sensory and motor cortices.* Two independent sections to be photographed were selected pseudo-randomly from those corresponding to the orientation planes spanning #560 - #620 of this same online atlas. Photomicrographs were taken of each of the following areas of interest: caudate nucleus (dorsal, medial, ventral; dCaud, mCaud and vCaud, respectively), putamen (dorsal, medial, ventral; dPut, mPut, and vPut, respectively), somatosensory cortex and motor cortex.

*Photomicrographs of the midline thalamus.* For the thalamus, two independent sections to be photographed were chosen pseudo-randomly from those corresponding to coronal orientation plane #780. Photomicrographs were taken of the following areas: the dorsal portion of the midline thalamus (encompassing the dorsal and ventral portions of the paraventricular nucleus of the thalamus; ThalPVd and ThalPVv, respectively), the ventral portion of the midline thalamus (encompassing the region of the midline thalamus just dorsal to the roof of the third ventricle, in the region of the Rhomboid and Reuniens nuclei; ThalRhRe), and the posterior hypothalamus, dorsal and ventral parts (pHYPdp, pHYPvp, respectively). For all areas (prefrontal cortex, striatum, and thalamus), one photomicrograph of each region of interest was taken from each of the two pseudo-randomly chosen sections. All digital photomicrographs were coded for subsequent blinded quantification of c-Fos labeling.

#### 2.2.4 Histological landmarks used for section sampling

*Prefrontal cortex (Cano-Ramirez and Hoffman, 2017*): The landmarks of the regions of interest, and the area of each region sampled were the following: ACC, a horizontally-oriented rectangle (1.58 mm × 0.76 mm) centered adjacent to the tip of the corpus callosum; PL, a rectangle (1.26 × 0.61 mm) centered ventrally to the ACC and laterally to the corpus callosum; IL, a horizontal rectangle(1.17 × 0.44 mm) positioned next to the inferior part of the lateral ventricle and ventrally to PL; piriform cortex, a rectangle (1.31 × 0.48 mm) covering the most central part of the lateral piriform cortex; OFC, a horizontal rectangle (1.17 × 0.53 mm), centered horizontally on the dorsal bank of the olfactory sulcus; premotor cortex, a vertically-oriented rectangle (1.5 × 0.9 mm). *Striatum and motor/somatosensory cortex (Cano-Ramirez and Hoffman, 2018)*: The landmarks of the regions of interest, and the dimensions of each region sampled were the following: the caudate nucleus was divided in three sections, dCaud: a horizontally-oriented ellipse (1.53 × 1.28 mm) centered adjacent to the tip of the internal capsule; mCaud: a vertically oriented rectangle (0.63 × 1.59 mm) centered ventrally to the dorsolateral caudate and medially to the internal capsule; vCaud: a horizontally- oriented ellipse (1.06 × 1.15 mm) centered medially to the inferior part of the internal capsule and lateral to the ventral-most extent of the lateral ventricle. The putamen was also divided in three sections; dPut: a vertically-oriented rectangle (1.0 × 1.6 mm) centered ventral to the corpus callosum and situated between the internal and external capsules; mPut: a vertically-oriented ellipse (1.41 × 1.14 mm) centered ventrally to the dorsolateral putamen and lateral to the internal capsule; vPut: a horizontally-oriented ellipse (1.35 × 1.55 mm) centered laterally to the inferior part of the internal capsule and dorsally to the anterior commissure. The areas sampled from the motor cortex and somatosensory cortex were vertically-oriented rectangles (0.54 mm x a variable length, the latter corresponding to the distance from the cortical surface to the corpus collosum). *Midline thalamus:* The areas sampled from the midline thalamus were as follows: ThalPVd, oval-shaped sampling areas (0.53 mm × 0.76 mm), oriented vertically and placed bilaterally just below the habenular nucleus, with the medial edge abutting the wall of the medial lateral ventricle; ThalPVv, rectangular sampling area (0.47 mm × 0.66 mm) positioned just below the oval sampling regions described above, and centered along the midline; ThalRhRe was the sum two sampling areas, a horizontally-oriented rectangular area (1.47 mm × 0.50 mm), its bottom edge abutting the roof of the third ventricle and centered with along the midline, and a square sampling area (0.90 mm × 0.90 mm), its bottom edge abutting the top of the rectangular area just mentioned, and centered along the midline; pHYPd, the sum of two vertically-oriented rectangles (0.76 mm × 1.37 mm) positioned bilaterally, their medial edges abutting the walls of the third ventricle; pHYPv, a square sampling region (1.42 mm × 1.42 mm), its top edge centered along the midline and abutting the floor of the third ventricle.

#### 2.2.5 Quantification of c-Fos label

The photomicrographs were coded, and c-Fos labeling was quantified by an observer blinded to the identity of the corresponding animal. Thus, each photomicrograph was cropped to include only the region of interest based on defined landmarks described above, and labelled cells within each region were counted in an automatic and unbiased manner using the software ImagePro version 5.1 (parameters: area 75–600, aspect 1–3). After quantifying the c-Fos label, the photomicrographs were decoded and the cell counts derived from the 2 sections per animal were summed, and c-Fos labeling density was calculated as the number of labeled cells per mm2.

#### 2.2.6 Behavior Analysis

Behaviors were quantified by means of observing video recordings (t = 0–30 min, encompassing the time during which the animal had access to straw) from each of the 5 animals. A datasheet was used in order to register the times (taken from the clock display on the videorecording) at which the animal performed each of the following sequential behaviors that comprise a “straw carrying cycle”: 1) began collecting straw; 2) entered nest box while carrying straw; 3) exited nest box; 4) began collecting straw again. Additional behaviors were noted: 5) “digging”, or vigorously scratching the floor of the nest box; 6) “deposit”, where all the straw carried in the mouth was deposited inside the nest box (infrequently, the rabbit exited the nest box while still carrying straw in her mouth). From this datasheet, latencies (with respect to t = 0 s) to enter nest box, dig, and deposit were calculated, along with the total number of straw carrying cycles/digs/deposits expressed across t = 0–30 min, and the number of cycles/digs/deposits expressed across t = 0–15 min, and across t = 16–30 min. For each independent cycle, the time spent collecting straw was calculated as time of #2 (see above) minus time of #1. Likewise, the time spent inside the nest box was calculated as the time of #3 minus the time of #2. Cycle duration was calculated as time of #4 minus time of #1. From these calculations, the mean time spent collecting per cycle, mean time spent inside the nest box per cycle, and the mean cycle duration were calculated, along with the standard deviations for each of these means.

#### 2.2.7 Statistical analysis

JASP 0.11 software (freely available online; https://jasp-stats.org) was used. Exploratory Factor Analysis (EFA) with cluster rotation was applied. 2, 3, and 4 factor solutions were run, and factor loadings less than 0.5 are not shown.

## Ethics Statement

Throughout this work, animal care adhered to the Law for the Protection of Animals (Mexico) and guidelines established by the National Institutes of Health for the care and use of Laboratory animals (NIH Publications No. 8023, revised 1978).

## CRediT Author Statement

**Hugo Cano-Ramírez:** Conceptualization, Methodology, Data Curation and Investigation. **Lorena Paola Pérez Martínez:** Conceptualization, Methodology, Data Curation and Investigation. **Kurt L. Hoffman:** Conceptualization, Resources, Formal Analysis, Writing, Supervision.

## Declaration of Competing Interest

The authors declare that they have no known competing financial interests or personal relationships which have, or could be perceived to have, influenced the work reported in this article.

## Appendix Supplementary materials

Image, application 1

Image, application 2
