# Supplementary material for: Dataset on c-Fos expression within components of corticostriatal thalamocortical circuits during the expression of a compulsive-like behavior in the female rabbit: Brain-behavior relationships
Source: Data Brief. 2020 Dec 30;34:106696. doi: 10.1016/j.dib.2020.106696 (PMC7807206; doi:10.1016/j.dib.2020.106696)
Supplement: Supplementary file 2 [file mmc2.docx]

1) Brain region data, 2-factor solution

| **Factor Loadings** | | | | | | | |
| --- | --- | --- | --- | --- | --- | --- | --- |
|  | | **Factor 1** | | **Factor 2** | | **Uniqueness** | |
| ACC |  | . |  | 0.604 |  | 0.500 |  |
| IL |  | . |  | 0.797 |  | 0.394 |  |
| Motor |  | 0.721 |  | 0.505 |  | 0.058 |  |
| OFC |  | . |  | 1.001 |  | 0.028 |  |
| PL |  | . |  | 0.923 |  | 0.048 |  |
| Piriform |  | -0.533 |  | 0.918 |  | 0.099 |  |
| Premotor |  | . |  | 0.858 |  | 0.127 |  |
| Somatosensory |  | 0.544 |  | . |  | 0.449 |  |
| ThalPVd |  | 0.788 |  | . |  | 0.412 |  |
| ThalPVv |  | 1.013 |  | . |  | 0.003 |  |
| ThalRhRe |  | 0.633 |  | . |  | 0.615 |  |
| dCaud |  | 0.736 |  | 0.502 |  | 0.034 |  |
| dPut |  | 0.690 |  | 0.549 |  | 0.047 |  |
| mCaud |  | . |  | 0.651 |  | 0.407 |  |
| mPut |  | 0.909 |  | . |  | 0.000 |  |
| vCaud |  | . |  | . |  | 0.783 |  |
| vHYPdp |  | 0.964 |  | . |  | -0.016 |  |
| vHYPvp |  | 1.008 |  | . |  | 0.031 |  |
| vPut |  | 0.618 |  | . |  | 0.638 |  |
|  | | | | | | | |
| *Note.*  Applied rotation method is cluster. | | | | | | | |

| **Factor Correlations** | | | | | |
| --- | --- | --- | --- | --- | --- |
|  | | **Factor 1** | | **Factor 2** | |
| Factor 1 |  | 1.000 |  |  |  |
| Factor 2 |  | -0.313 |  | 1.000 |  |
|  | | | | | |

| **Chi-squared Test** | | | | | | | |
| --- | --- | --- | --- | --- | --- | --- | --- |
|  | | **Value** | | **df** | | **p** | |
| Model |  | . |  | 134 |  | . |  |
|  | | | | | | | |

2) Brain región data, 4-factor solution

| **Factor Loadings** | | | | | | | | | | | |
| --- | --- | --- | --- | --- | --- | --- | --- | --- | --- | --- | --- |
|  | | **Factor 1** | | **Factor 2** | | **Factor 3** | | **Factor 4** | | **Uniqueness** | |
| ACC |  | . |  | . |  | . |  | 0.894 |  | 0.001 |  |
| IL |  | . |  | -0.529 |  | . |  | 0.635 |  | 0.001 |  |
| Motor |  | 0.702 |  | 0.556 |  | . |  | . |  | 0.001 |  |
| OFC |  | 0.929 |  | . |  | . |  | . |  | 0.001 |  |
| PL |  | 0.980 |  | . |  | . |  | . |  | 0.001 |  |
| Piriform |  | 0.733 |  | . |  | . |  | . |  | 0.001 |  |
| Premotor |  | 1.011 |  | . |  | . |  | . |  | 0.001 |  |
| Somatosensory |  | . |  | . |  | 0.837 |  | . |  | 0.001 |  |
| ThalPVd |  | . |  | 1.134 |  | . |  | . |  | 0.002 |  |
| ThalPVv |  | . |  | 0.909 |  | . |  | . |  | 0.001 |  |
| ThalRhRe |  | . |  | 0.930 |  | -0.524 |  | . |  | 0.001 |  |
| dCaud |  | 0.673 |  | . |  | . |  | . |  | 0.001 |  |
| dPut |  | 0.625 |  | . |  | . |  | . |  | 0.001 |  |
| mCaud |  | 1.019 |  | . |  | . |  | . |  | 0.001 |  |
| mPut |  | . |  | 0.753 |  | . |  | . |  | 0.001 |  |
| vCaud |  | . |  | . |  | . |  | 1.035 |  | 0.002 |  |
| vHYPdp |  | . |  | 0.705 |  | . |  | . |  | 0.001 |  |
| vHYPvp |  | . |  | 0.737 |  | . |  | . |  | 0.001 |  |
| vPut |  | . |  | . |  | 0.978 |  | . |  | 0.002 |  |
|  | | | | | | | | | | | |
| *Note.*  Applied rotation method is cluster. | | | | | | | | | | | |

| **Factor Correlations** | | | | | | | | | |
| --- | --- | --- | --- | --- | --- | --- | --- | --- | --- |
|  | | **Factor 1** | | **Factor 2** | | **Factor 3** | | **Factor 4** | |
| Factor 1 |  | 1.000 |  |  |  |  |  |  |  |
| Factor 2 |  | 0.238 |  | 1.000 |  |  |  |  |  |
| Factor 3 |  | 0.207 |  | 0.492 |  | 1.000 |  |  |  |
| Factor 4 |  | 0.300 |  | -0.084 |  | 0.172 |  | 1.000 |  |
|  | | | | | | | | | |

| **Chi-squared Test** | | | | | | | |
| --- | --- | --- | --- | --- | --- | --- | --- |
|  | | **Value** | | **df** | | **p** | |
| Model |  | 0.000 |  | 101 |  | 1.000 |  |
|  | | | | | | | |

3) Brain region and behavior data, 2-factor solution

| **Factor Loadings** | | | | | | | |
| --- | --- | --- | --- | --- | --- | --- | --- |
|  | | **Factor 1** | | **Factor 2** | | **Uniqueness** | |
| ACC |  | . |  | 0.814 |  | 0.328 |  |
| Cycles15 |  | 0.771 |  | . |  | 0.409 |  |
| Cycles30 |  | 0.995 |  | . |  | 0.010 |  |
| IL |  | . |  | 0.935 |  | 0.026 |  |
| InsideMax |  | -0.809 |  | 0.525 |  | 0.147 |  |
| InsideMean |  | -0.829 |  | 0.616 |  | 0.025 |  |
| Motor |  | 0.775 |  | 0.545 |  | 0.026 |  |
| OFC |  | . |  | 0.836 |  | 0.290 |  |
| OutsideMean |  | . |  | -0.973 |  | 0.061 |  |
| PL |  | . |  | 0.822 |  | 0.197 |  |
| Piriform |  | . |  | 0.663 |  | 0.475 |  |
| Premotor |  | . |  | 0.739 |  | 0.265 |  |
| Somatosensory |  | . |  | 0.649 |  | 0.402 |  |
| ThalPVd |  | 0.633 |  | . |  | 0.594 |  |
| ThalPVv |  | 0.952 |  | . |  | 0.084 |  |
| ThalRhRe |  | 0.828 |  | . |  | 0.262 |  |
| dCaud |  | 0.742 |  | 0.603 |  | 0.005 |  |
| dPut |  | 0.651 |  | 0.685 |  | 0.028 |  |
| mCaud |  | 0.512 |  | . |  | 0.455 |  |
| mPut |  | 0.849 |  | . |  | 0.046 |  |
| vCaud |  | . |  | 0.702 |  | 0.465 |  |
| vHYPdp |  | 0.891 |  | . |  | 0.044 |  |
| vHYPvp |  | 0.817 |  | . |  | 0.296 |  |
| vPut |  | . |  | . |  | 0.814 |  |
|  | | | | | | | |
| *Note.*  Applied rotation method is cluster. | | | | | | | |

| **Factor Correlations** | | | | | |
| --- | --- | --- | --- | --- | --- |
|  | | **Factor 1** | | **Factor 2** | |
| Factor 1 |  | 1.000 |  |  |  |
| Factor 2 |  | 0.010 |  | 1.000 |  |
|  | | | | | |

| **Chi-squared Test** | | | | | | | |
| --- | --- | --- | --- | --- | --- | --- | --- |
|  | | **Value** | | **df** | | **p** | |
| Model |  | 0.000 |  | 229 |  | 1.000 |  |
|  | | | | | | | |

4) Brain region and behavior data, 4-factor solution

| **Factor Loadings** | | | | | | | | | | | |
| --- | --- | --- | --- | --- | --- | --- | --- | --- | --- | --- | --- |
|  | | **Factor 1** | | **Factor 2** | | **Factor 3** | | **Factor 4** | | **Uniqueness** | |
| ACC |  | . |  | 0.820 |  | 0.633 |  | . |  | 0.001 |  |
| Cycles15 |  | 0.576 |  | -0.890 |  | . |  | . |  | 0.001 |  |
| Cycles30 |  | . |  | -0.627 |  | . |  | . |  | 0.001 |  |
| IL |  | 0.514 |  | 0.711 |  | . |  | . |  | 0.001 |  |
| InsideMax |  | . |  | 0.965 |  | . |  | . |  | 0.001 |  |
| InsideMean |  | . |  | 0.844 |  | . |  | . |  | 0.001 |  |
| Motor |  | 0.695 |  | . |  | 0.501 |  | . |  | 0.001 |  |
| OFC |  | 1.003 |  | . |  | . |  | . |  | 0.001 |  |
| OutsideMean |  | -0.749 |  | . |  | . |  | . |  | 0.001 |  |
| PL |  | 1.021 |  | . |  | . |  | . |  | 0.001 |  |
| Piriform |  | 0.828 |  | . |  | . |  | . |  | 0.001 |  |
| Premotor |  | 1.029 |  | . |  | . |  | . |  | 0.001 |  |
| Somatosensory |  | . |  | . |  | . |  | 0.745 |  | 0.001 |  |
| ThalPVd |  | . |  | . |  | 1.161 |  | . |  | 0.002 |  |
| ThalPVv |  | . |  | . |  | 0.789 |  | . |  | 0.001 |  |
| ThalRhRe |  | . |  | -0.593 |  | 0.621 |  | . |  | 0.001 |  |
| dCaud |  | 0.669 |  | . |  | . |  | . |  | 0.001 |  |
| dPut |  | 0.646 |  | . |  | . |  | . |  | 0.001 |  |
| mCaud |  | 0.981 |  | . |  | . |  | . |  | 0.001 |  |
| mPut |  | . |  | . |  | 0.753 |  | . |  | 0.001 |  |
| vCaud |  | . |  | 1.022 |  | 0.510 |  | . |  | 0.001 |  |
| vHYPdp |  | . |  | . |  | 0.703 |  | . |  | 0.001 |  |
| vHYPvp |  | . |  | . |  | 0.794 |  | . |  | 0.001 |  |
| vPut |  | . |  | . |  | . |  | 0.903 |  | 0.002 |  |
|  | | | | | | | | | | | |
| *Note.*  Applied rotation method is cluster. | | | | | | | | | | | |

| **Factor Correlations** | | | | | | | | | |
| --- | --- | --- | --- | --- | --- | --- | --- | --- | --- |
|  | | **Factor 1** | | **Factor 2** | | **Factor 3** | | **Factor 4** | |
| Factor 1 |  | 1.000 |  |  |  |  |  |  |  |
| Factor 2 |  | 0.076 |  | 1.000 |  |  |  |  |  |
| Factor 3 |  | 0.316 |  | -0.320 |  | 1.000 |  |  |  |
| Factor 4 |  | 0.196 |  | -0.022 |  | 0.350 |  | 1.000 |  |
|  | | | | | | | | | |

| **Chi-squared Test** | | | | | | | |
| --- | --- | --- | --- | --- | --- | --- | --- |
|  | | **Value** | | **df** | | **p** | |
| Model |  | 0.000 |  | 186 |  | 1.000 |  |
|  | | | | | | | |
